# “They wanted to, but they just couldn’t get there”: GBA + implementation and gaps during the COVID-19 pandemic in Canada

**DOI:** 10.1186/s12939-025-02522-2

**Published:** 2025-05-27

**Authors:** Muhammad Haaris Tiwana, Lara Hollmann, Julia Smith

**Affiliations:** Faculty of Health Sciences, Simon Fraser University, Burnaby, Canada

**Keywords:** Canada, Equity, Policy analysis, Pandemic, COVID-19, Priority populations, Gender based analysis

## Abstract

**Purpose:**

To improve understanding of the barriers and enablers to implementing gender and intersectional analysis during the COVID-19 pandemic in Canada.

**Methods:**

We conducted a policy document analysis (*n* = 70) of equity-focused policies of the Canadian government published between March 2020 and August 2023. This analysis was complemented with 16 semi-structured key informant interviews with federal policy actors and leadership of civil society organizations.

**Results:**

Pandemic policy documents demonstrated multiple commitments to address pandemic related inequities, with key informants describing collaborative approaches to implementing these policies, but also limits in terms of the urgent and diffused nature of pandemic response. Implementation gaps related to accessible information, health services and vaccinations were noted and attributed to a reliance on civil society actors who lacked sufficient and sustainable resources, and the behaviors of priority populations whose capacity to comply was limited by the same inequities the policies sought to address.

**Conclusion:**

The Canadian federal government made concerted efforts to address the needs of a range of priority populations and equity issue areas within its pandemic response, with mixed results. Having a pre-established framework to guide implementation and related relationships overcame some of the urgency challenges related with pandemic response. However, implementation gaps reflected preexisting inequities shaped by broader economic, social and political systems which were infrequently addressed in pandemic policies. There is a need for greater understanding of policy implementation gaps during emergency and crisis response.

**Supplementary Information:**

The online version contains supplementary material available at 10.1186/s12939-025-02522-2.

## Introduction

Research on infectious disease events, public health emergencies and pandemics [1], has highlighted the impact of social determinants of health, which exacerbate the onset, course and outcome of infectious diseases among equity-deserving populations [2, 3]. Concurrently, pandemic response policies can act as structural determinants of health, alongside biological determinants, to influence not only direct health outcomes but also secondary outcomes - defined as those that result from measures implemented to mitigate the direct health consequences - such as economic hardship and increased risk of violence [4, 5]. Existing literature extensively documents how these secondary effects exacerbate pre-existing vulnerabilities within society, particularly along lines of gender, race, class, socioeconomic status, and other intersecting factors, resulting in calls for pandemic response that centers the needs of those most vulnerable [6, 7]. However, beyond pointing out such impacts, and gaps within existing policies, there is little analysis of attempts to mitigate the unequal secondary effects of pandemic response.

Canada presents one example of an intentionally equity focused pandemic response. The Canadian federal government has been explicitly committed to implementing gender-based and intersectional policies through the mainstreaming of its Gender-Based Analysis Plus (GBA+) framework across policy spheres since 2011, and applied this approach to the COVID-19 pandemic response [8, 9]. This paper aims to better understand the barriers and enablers experienced implementing equity-based policies during the COVID-19 pandemic in Canada. We apply established theories of policy implementation to assess what equity considerations were reflected in the Canadian federal government COVID-19 policy response, using a content analysis of policy documents [10]. We then draw on key informant interviews with federal government and civil society actors, to explore how these played out in the pandemic context through analysis of select implementation gaps. While our paper is specific to Canada, findings point to the challenges of addressing the unequal effects of pandemics on priority populations more broadly, and the need for further analysis of implementation gaps within crisis policy responses in particular.

### Conceptualizing pandemic policy and implementation gaps

Our analysis is informed by previous conceptual frameworks on policy implementation gaps. Ansell et al. define policy implementation gaps as “the emergence of a significant gap between the planned outputs and outcomes of public policy” [11]. Research from political science and public administration scholars recognizes factors that shape policy implementation are complex and multilevel, reflecting the dynamics of overarching governance systems, and are constrained by the political economy in which they are implemented [12]. Recent literature also challenges assumptions of linear policy processes, recognizing that policies are implemented in complex contexts and that policies rarely are absolute failures or successes, but fall somewhere along a spectrum [13]. One common framework argues there are four main reasons for policy failure: overly optimistic expectations, implementation in dispersed governance, inadequate collaborative policymaking and vagaries of the political life cycle [14]. This builds on previous analyses that attribute policy failures to top-down decision-making, bottom-up influences (i.e. street level bureaucrats who are responsible for implementation but may not have the will, skills or resources required), outside-in factors (i.e. unintended and unforeseen behavior of target groups), or simply being badly designed [15].

These approaches to conceptualizing policy implementation gaps do not consider the specific characteristics of pandemic, or crisis, policymaking. For example, the uncertainty of a crisis may reduce pressure related to expectations and inspire unique governance arrangements [16]. Not only is the typical cycle of policy development (agenda setting, formulation and implementation) conflated during emergency response, political pressure is atypical as political opponents often put aside differences during crisis [17]. The top-down nature of crisis management can overcome some challenges within dispersed governance frameworks, but also reduces opportunities for collaboration. Indeed, command and control style decision-making is often relied on during emergency response, the theory behind which draws on military traditions, as opposed to public policy approaches [18, 19]. Policy-implementing actors are expected to follow orders to reduce the number of uncertainties. However, they may lack the necessary resources—particularly in the short term—or the skills to carry out directives effectively. Additionally, implementors often face unpredictable behaviors of population groups in rapidly changing contexts. The unique nature of pandemic policy making raises questions about established understandings of policy implementation gaps in such situations; we outline these dynamics in Table 1. In what follows, we aim to apply established conceptualizations of policy implementation to pandemic response in Canada to better to better understand the barriers and enablers experienced implementing equity-based policies during the COVID-19 pandemic in Canada.

**Table 1:** Conceptual framework

Established reasons for policy gaps/failures	Pandemic policy dynamics
Overly optimistic expectations	Expectations tempered by accepted uncertainty
Dispersed governance	Command and control style governance
Inadequate collaboration	Urgency reduces opportunities for collaboration
Political lifecycle	Less relevant
Top-down decision-making	Heightened
Bottom-up influences	Under resourced
Outside in factors	Unpredictable
Poor design	Urgency trumps design

### Pandemic policymaking in the Canadian context

As noted above, Canada’s pandemic response explicitly aimed to incorporate gender and intersectional analysis through the application of its specific GBA + framework. Managed by Women and Gender Equality Canada (WAGE), GBA + serves as a multifaceted analytical framework to evaluate and shape inclusive policies and programs across the federal government. It considers not only biological and gender differences but also factors such as age, disability, ethnicity, geography, and religion [20]. Applications of GBA + at the time of writing are informed by the 2016 GBA + action plan, which aimed to strengthen governance networks to address systemic inequalities and enhance training for government officials [21], as well as the Canadian Gender Budgeting Act, mandating the integration of GBA + into all new annual budget measures and tax expenditures [22]. Since its implementation, GBA + has faced criticism for perceived bureaucratic constraints, limited visibility beyond social policy sectors and provincial implementation, and its narrow application scope [23].

GBA+, and overall policy statements purporting an equity-based pandemic response, reflect federal policy commitments. The Canadian federal system allocates powers and responsibilities between the federal agencies, provincial governments and regional/municipal authorities, resulting in a multi-level policy jurisdiction [24]. While the provincial governments have jurisdictional authority over healthcare and most social programs, the COVID-19 pandemic saw the federal government take a lead role in health, social and economic policymaking, providing, for example, 86% of funding for the COVID-19 response [25]. Implementation, however, was often left to provincial agencies and regional health authorities, making it highly dependent on local context. Here we focus on federal level policy implementation, while noting the role of other non-governmental policy actors.

As multiple studies point out, policy implementation is dependent on resources. In this paper, we do not analyze funding allocation related to these policies, priority populations and issue areas, as that would entail another study, which has already been done to some degree by others [25, 26]. However, it is helpful to note that Canada provided in total nearly 19% of GDP to the COVID-19 response, more than most comparable OECD countries. The lion’s share of direct spending—$366 billion, or 86%—came from the federal government [25]. As these figures demonstrate a notable investment in the COVID-19 response by the Canadian government and, as noted analysis of how these resources were distributed is available elsewhere, we do not include detailed resource allocation on our analysis of policy implementation here.

## Methods

### Document identification

To first understand what equity-based policies were implemented during the COVID-19 pandemic, we began a two-step analysis beginning with a content analysis of policy documents [10]. Policy documents were defined as any documentation providing details on policy choices and priorities, including legislative policy documents, press releases, speeches, online information from government sources. For document identification, two authors began searching for any policy documents related to the COVID-19 pandemic (i.e. mentioned COVID or synonyms such as ‘pandemic’) between March 2020 and August 2023 (inclusive), much of which are publicly available and were sourced through systematic searches of federal institutions’ websites, media articles, peer-reviewed policy assessments, as well as targeted google searches. Additionally, existing databases were cross-referenced to ensure comprehensive coverage, including sources like the WHO Public Health & Social Measures, Canadian Institute for Health Information, StatsCan, Oxfam Canada, and the Canadian Centre for Policy Alternatives. We limited our inclusion to those policies directed at individuals or priority populations (as opposed to businesses or sectors as a whole). Where there was uncertainty regarding if a document should be included, the authors consulted with one another and the third author. This process resulted in the identification of 185 federal policy documents.

### Document inclusion criteria for equity analysis

**Fig. 1:**
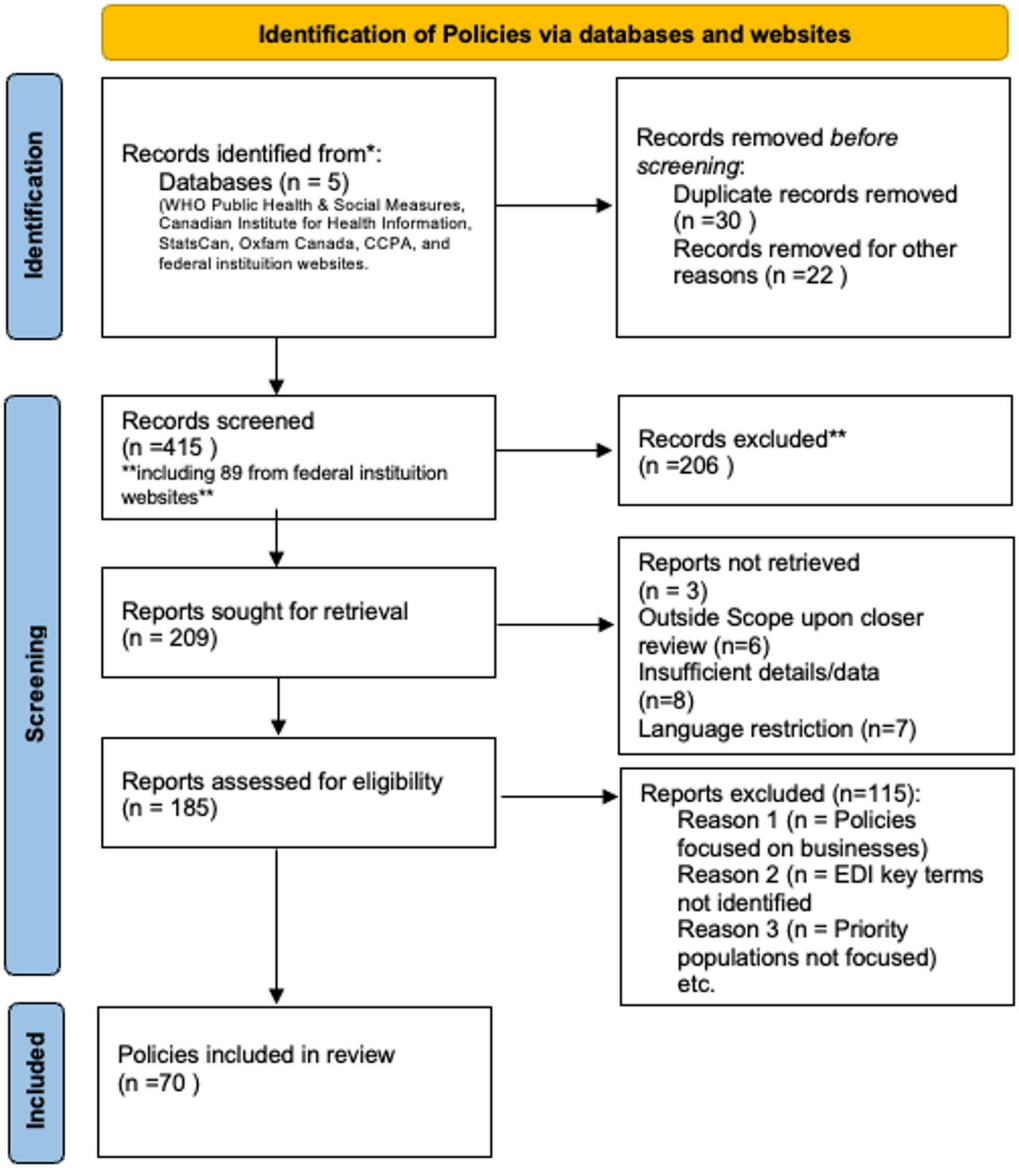
Prisma Flow diagram of identification of policies

For document inclusion in the equity-focused analysis, the 185 documents were then searched using key terms from both federal and provincial Equity, Diversity and Inclusion (EDI) glossaries and health units’ language guides [27–29]. These terms covered priority populations such as women, sexual gender minorities, LGBTQ+, racialized persons, Indigenous peoples, migrants (including asylum seekers and refugees), those with low-income, people with disabilities, and older adults, adolescents and children. They also include key issue areas relevant to priority populations such as interpersonal violence, digital divide, accessibility (Supplementary file 1). Only those documents which included one or more of these terms were included in our analysis, based on the assumption they reflected equity related polices goals or the integration of GBA + into pandemic policy. This resulted in 70 federal policy documents for analysis (Fig. 1).

### Content analysis

In order to identify what equity considerations were included and prioritized in the federal policy response to the COVID-19 pandemic, we then conducted quantitative content analysis of these documents, tallying mentions of key populations and equity issue areas listed in glossaries noted above, as well as where and by who the policies were issued. In addition, we analyzed where and what priority populations and issue areas were mentioned in the same document (Supplementary file 1). Content related to priority populations and issues areas (i.e. where search terms were found) was then extracted and grouped (i.e. all content including mention of caregivers was extracted into one spreadsheet). Two authors analyzed this content to identify key policy initiatives, as well as illustrative quotes. All authors reviewed this content and discussed key initiatives and illustrative quotes until agreement was reached.

### Key informant interviews

We then conducted semi-structured key informant interviews with federal policy actors engaged in pandemic policy development and leadership of civil society organizations (CSO) that represent and/or serve priority populations and engaged with federal policy actors either through funding or other relationships. We included these CSOs as a means to incorporate expertise from those with knowledge of the experiences, needs and challenges of priority populations during the pandemic; as actors that often serve as intermediaries between priority populations and government, including implementing government policies through funding and other relationships; and as those with policy expertise and knowledge that are outside government and therefore able to provide a more critical perspective. Ethics approval was provided by the Office of Research Ethics at Simon Fraser University. Key informants were purposively sampled by considering the priority populations listed in our search terms and seeking representation from each through existing contacts and internet searches, with subsequent snowball sampling. Sixteen semi-structured interviews were conducted, eight from federal government agencies and eight from civil society organizations. Those interviewed were at the director level in their respective institutions. The federal agencies included Public Safety Canada, the Public Health Agency of Canada, Employment and Social Development Canada, Immigration, Refugees and Citizenship Canada, Health Canada, Statistics Canada, and Women and Gender Equality Canada. The civil society organizations focused on issues related to racism, gender equality, cultural safety, sexual identity and orientation, immigration status and protections, socioeconomic and job precarity, and age-related barriers. All interviews were conducted over Zoom and lasted 30 to 60 min. They followed a semi-structured guide (Supplementary file 2) regarding equity considerations within the COVID-19 context, priorities within the pandemic response, impact of subsequent policy responses on priority populations served, and successes and challenges of working within the response.

With consent, the interviews were recorded and transcribed. The interviews were analyzed in NVivo using framework analysis [30]. This involved two reviewers independently and inductively generating codes, followed by a comparison and grouping of these codes. Consensus was achieved through an iterative process of refining codes and mapping them into themes and sub-themes until no additional codes were left (Table 2) [31]. To ensure consistency in interpretation and further refine the analysis, the two reviewers initially coded two sets of transcripts, before independently coding all transcripts. This analysis was combined with the policy document analysis to consider factors effecting equity related pandemic policy implementation and identify select policy topics for in-depth analysis. Considering the limited sample size, insights from interviews are meant to be illustrative and prompting further inquiry, as opposed to generalizable and conclusive. In the discussion we bring the analysis of pandemic policies, implementation and gaps together with the conceptual framework around policy implementation gaps and pandemic response outlined above.

**Table 2:** Themes and sub-themes from interview analysis

Themes	Sub-themes
Policy Implementation	Established GBA + practices Funding Collaborations Bureaucracy
Access to Information	Language Plain Language Culture Understanding public health guidance Filling information gaps
Access to Healthcare	Mental healthcare Transportation Costs
Access to Vaccines	Physical barriers Time and resources Information CSOs facilitating access

## Results

### Equity consideration in federal policy documents

The analysis of equity considerations in federal policy documents during the first three years of the COVID-19 pandemic presents a diverse array of policy documents across different governmental bodies, with Employment and Social Development Canada (*n* = 15), Public Health Agency of Canada (*n* = 11), and Indigenous Services Canada (*n* = 10) being the most common issuers (Table 3). This distribution underscores both the multi-agency approach taken by the Canadian government to address equity concerns, as well as the greater role of some agencies in implementing GBA+. The significant presence of agencies such as Public Health Agency of Canada and limitedly Health Canada also highlights the prioritization of equity considerations within pandemic policy.

**Table 3:** Amount of policy documents identified by issuing agency

Issuing Agency	Amount
Canada Revenue Agency	2
Canadian Heritage	2
Correctional Services of Canada	1
Department of Finance Canada	2
Employment and Social Development Canada	15
Government of Canada	2
Health Canada	3
Indigenous Services Canada	10
National Advisory Committee on Immunizations	1
National Defense	4
Prime Minister	8
Public Health Agency of Canada	11
Public Safety Canada	6
Public Service & Procurement Canada	1
WAGE	2
**Grand Total**	**70**

### References to priority populations

Table 4, presents priority populations mentioned in policy documents, highlighting the government’s efforts to meet the needs of marginalized groups with various demographic and socio-economic factors taken into account. The notable mention of Indigenous Peoples reflects that Indigenous Peoples in Canada experience disproportionate health risk due to colonization which has resulted in lack of access to culturally appropriate and accessible health services, as well as social and economic marginalization. In addition, the federal government has legislative authority in relation to Indigenous reserve lands, and Canada’s Northwest, Nunavut and Yukon territories (where the majority of the population is Indigenous) [32].

**Table 4:** Priority population addressed by policy documents

Priority population mentioned in policy documents	Amount
Caregivers	2
English as Second/Foreign language speakers	4
Families	6
Health Care Workers	1
Immigrants	5
Indigenous People	28
LGBTQI+	8
Minors	10
Older adults	15
People with disability	9
Racialized populations	17
Religious minorities	2
Socio-economic status	15
Students	2
Unpaid care workers	1
Women	14
No population specified	6
**Grand total**	**70**

### Issue areas

Table 5 notes issue areas addressed by policy documents, revealing topics ranging from accessibility and diversity to mental health and public safety. The most frequently addressed issues in the policy documents were accessibility (36 mentions), equity (20), and marginalization (12), indicating a strong emphasis on improving access to services and addressing structural inequities. Accessibility, the most common equity term used in documents, refers to access to health and social services. For example, the government increased investments in virtual health, as a means to improving access to health services [33]. The Prime Minister also announced supports for access to basic necessities for the northern communities, stating “…. we are working with the territories and Indigenous partners to address the unique needs of northern communities as they respond to COVID-19. Together, we will make sure that Northerners can access the food, supplies, health care, and services they need during this challenging time” [34].

**Table 5:** Issue areas addressed by policy documents (multiple mentions possible)

Issue areas addressed by policy document	Amount
Accessibility	36
Colonialism	2
Discrimination	4
Diversity	5
Equity	20
Gender	5
Intersectionality	4
Marginalization	12
Mental health	8
Privilege	1
Public Safety	6
Sexual orientation	4
Trauma	5
**Grand total**	**112**

### Overlap between priority populations and issue areas

**Fig. 2:**
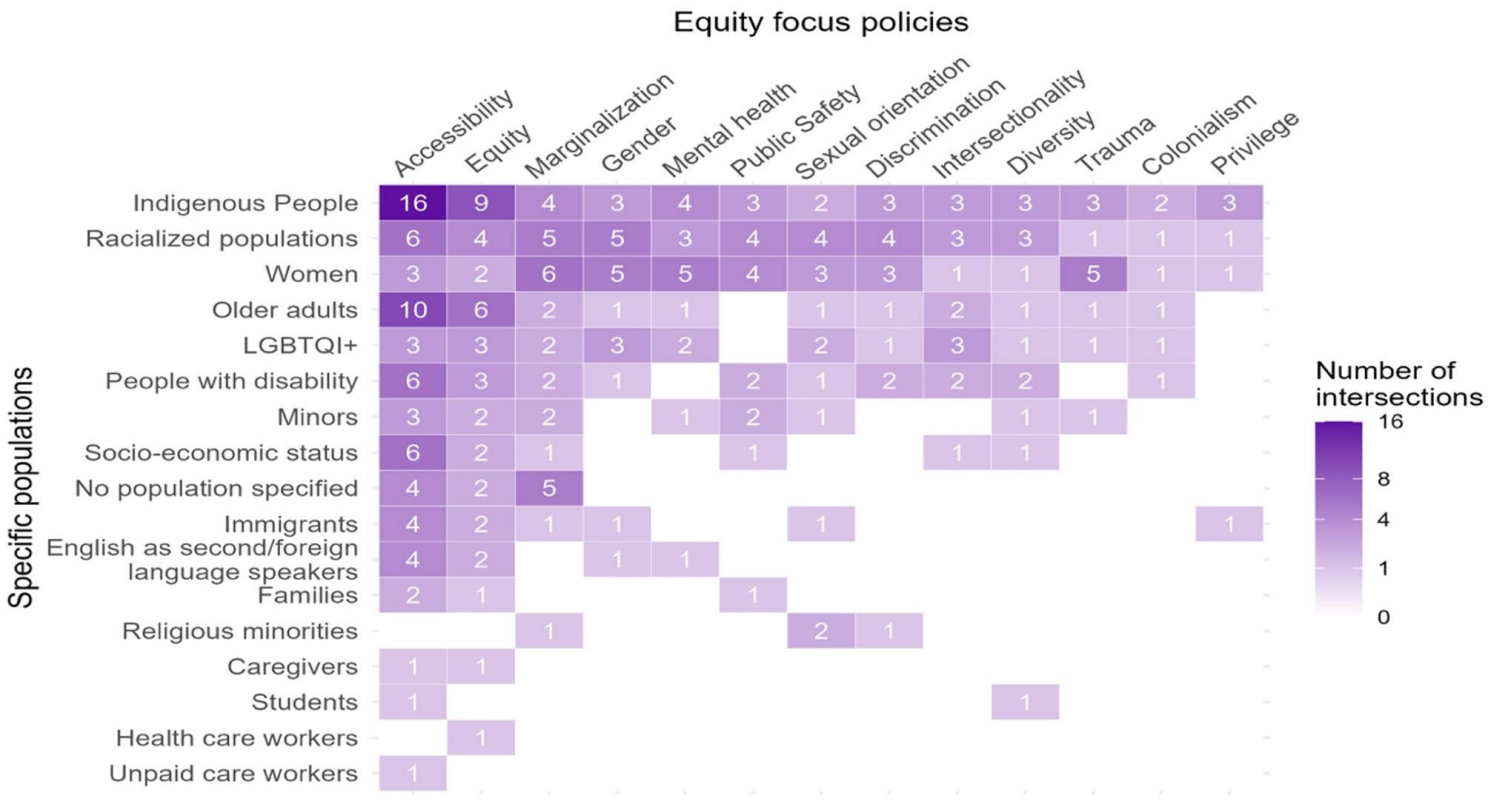
Intersection heat map of priority population and issue areas

Analysis of policy documents reveals overlaps between priority populations and issue areas (Fig. 2). For instance, policies that mention Indigenous Peoples were also likely to mention colonialism, and trauma, noting the compounded challenges faced by Indigenous communities. In recognition of how such factors might increase the need for health services during the pandemic, the government supported the public health response in Quebec by deploying the Canadian Armed Forces to assist remote Inuit communities and allocated $285.1 million to support COVID-19 responses in Indigenous communities [35]. While significant, this funding must be considered against the longstanding systemic health inequities faced by Indigenous communities, including limited access to healthcare infrastructure and higher rates of chronic diseases, which exacerbate vulnerabilities during a pandemic [36, 37]. Similarly, policies reviewed that mentioned racialized populations note they experienced intersecting issues related to discrimination, gender prejudice, and socioeconomic disparities, emphasizing the need for interventions to address systemic inequities [38].

Figure 2 depicts a focus on accessibility, equity, Indigenous peoples, women and older adults. There were few mentions of the structural determinants of inequities, such as colonialism and privilege, and of priority populations such as caregivers and students. These findings point to both priorities within the federal response and preferred terminology. Intersectionality (a term developed out of black feminist thinking that refers to the study of intersecting systems of power and oppression) is mentioned across the six most frequently noted priority populations, suggesting a recognition that these groups experience multiple inequities related to social position [39].

### Pandemic policy implementation

This section presents key themes from the interviews with federal government and civil society actors regarding the implementation of equity-based policies during the COVID-19 pandemic. The findings are organized around four main areas: pre-existing collaborative mechanisms, the role of civil society organizations, the influence of bureaucratic processes and urgency on equity efforts, and challenges related to funding and sustainability.

Interviewees noted that pre-existing collaborative mechanisms, based on GBA+, proved valuable in implementing equity-based responses during the pandemic. One federal interviewee explained, “We have a lot of mechanisms already set up that facilitate that ongoing loop of feedback and information. You know, they’re not– none of them are perfect, obviously. But I think that a lot of the mechanisms that were in place previously facilitated additional collaboration with civil society during the pandemic” (4 F-KII). This pre-established infrastructure allowed for smooth transition of collaborative efforts during the crisis. CSOs also noted the benefits of having existing relationships with government actors and each other, including help with funding, expanding their reach to a broader community base and having up to date knowledge of federal policy and decision-making (6 C-KII and 2 F-KII). These relationships facilitated policy delivery, aligning priorities and messaging across government and CSOs, as well as identifying synergies to work together (7 C-KII).

The urgency of the pandemic allowed a circumvention of traditional bureaucratic barriers, enabling more agile responses. One informant described this shift: “Instead of having to jump through hoops, instead of having to go through red tape… you were able to be like, ‘We need this. And hospital, I need this from you. Can you give us this?’” (1 C-KII). This sense of urgency fostered a collaborative spirit and reduced bureaucratic delays: “That sense of crisis, you know, impending doom certainly pushes people to work together better. To dispense with some of the bureaucratic shittiness that’s there to be able to just get things done that needed to get done.” (1 C-KII).

Respondents also described the challenges of implementing equity-based responses during a public health crisis including lack of consistent application due to the urgency of pandemic response (2 F-KII). One respondent noted, “… the guidance is that GBA + must be considered at all levels of all of our work.. [but] things that move really fast that do not allow for the kind of extensive work that, you know, in an ideal world would be applied. Sometimes GBA + is applied a little bit last minute, especially when things are rushed” (1 F-KII). Many noted that urgency impedes the development of evidence-based GBA + policies, resulting in current political preferences having undue influence (4 F-KII). Others noted bureaucratic processes prevented timely access to data required to make evidence informed decisions (5 F-KII). CSOs noted that the rapid timeline inhibited meaningful consultation: “They’ll do one small focus group. That’s not very representative of what a given community would like” (2 C-KII).

Much federal funding aimed at addressing secondary effects was directed to CSOs, which were recognized as having the expertise and relationships to best support priority populations [40, 41]. Federal actors emphasized the importance of working with CSOs in order to access information about what was happening at the community level (4 F-KII). CSOs commented that federal funding provided to them was initially dispersed quickly (4 C-KII), facilitating rapid implementation, and needs of marginalized populations which previously would have not received the same level of attention were recognized (5 F-KII). However, all CSOs also noted funding was insufficient to meet needs, a complaint confirmed by a federal actor who noted many proposals they felt were worthy were not funded due to lack of resources (3 F-KII). CSOs also noted that initial government funding commitments were not maintained, and organizations struggled, after the initial year of the pandemic, to meet ongoing needs of constituents (4 C-KII). Others noted that the majority of government funding to CSOs went to large organizations. One example is the Emergency Community Services Fund of $350 million to support vulnerable Canadians, disseminated through three national organizations: Canadian Red Cross Society (~$80 M), United Way of Canada (~$120 M), and Community Foundations of Canada (~$75 M) [42]. Smaller organizations were often excluded from this funding unless they had connections with these national bodies [42]. One common need that the interviewees reiterated was that funding was less likely to cover general operational costs, such as existing staff cost, vehicles or software licenses; while these were not directly related to pandemic response, without these necessities CSOs could not implement COVID-19 specific programs (4 C-KII).

### Policy gaps: accessibility

In this section we draw on key informant interviews and the content of the policy documents, to analyze implementation gaps across three specific pandemic related policy areas: access to information, access to healthcare and access to vaccines. These three examples reflect priority policy areas, with accessibility the most mentioned issue area in the document analysis above, and as three topics that emerged across all key informant interviews. We use these examples to illustrate select implementation gaps.

#### Access to information

The majority of interviewees, both CSO (*n* = 6) and federal actors (*n* = 4), identified **language** as a key challenge to implement COVID-19 response measures for priority populations. In 2021, there were more than 200 languages spoken in Canada with more than 23% of the population considering another language than English or French as their native language [43]. Four policies, out of the 70 analyzed, supported or funded linguistically appropriate response measures. For example, the Public Health Agency of Canada funded four projects through the Immunization Partnership Fund to support vaccination uptake among populations disproportionately impacted by COVID-19 through targeted outreach and culturally relevant interventions [44]. Another project supported translations of information in Indigenous languages to support First Nation organizations that deliver community-based services in First Nation communities on-reserve as well as communities in the Northern territories [45]. A federal actor noted activities were conducted to facilitate access to information for newcomers to Canada who were not fluent in English or French through settlement services and newcomer language classes.

Despite such interventions, numerous CSO interviewees noted that early public health guidelines and information was not available more broadly in languages other than English and French, Canada’s official government languages, or at least translations were not reaching the necessary communities. One interviewee also raised concerns about the lack of American Sign Language (ASL) interpretations available (6 C-KII). Where translations were available, interviewees shared concerns about the possible inaccessibility of translations due to the type of language used. For example, a key informant reflected on past experiences working with Spanish-speaking communities from Latin America: “[An organization] had [pamphlets] translated into Spanish, but it was Spanish from Spain. Like this is like studying– like imagine almost English from Shakespeare’s time and workers are not going to understand it. […] You know, you can’t have like an expert from– like a Spanish speaker from Spain ‘’ (3 C-KII).

Interviewees also noted that much of the information provided often failed to consider cultural, linguistic, and socioeconomic differences, leading to gaps in understanding and compliance. Many CSOs highlighted the necessity of creating culturally relevant resources. For example, one interviewee raised concerns that most mental health resources for youth appeared to be designed primarily for the white population (6 C-KII).

Despite the fact that the Canadian government made commitments to publishing any content targeted at the public in plain language even before the pandemic [46], interviewees raised concerns that available information in English was not always written in an accessible manner. During the pandemic, the government invested in the development of a bilingual National Community of Practice to help community health agencies share resources and best practices across Canada [47]. However, key informants from both civil society and federal agencies noted many materials were not easily understood even by themselves (3 F-KII, 4 C-KII). The lack of plain and clear language information also impeded translations, with one CSO representative describing the challenge of “translating garbage to garbage.” (1 C-KII), due to lack of clarity in original communications.

Beyond demonstrating limitations in terms of implementing equity-based access to information policies, lack of translation and plain language communications likely inhibited the ability of priority populations to follow public health guidance and access support services designed to meet their needs. For example, one study with recent immigrant women found lack of awareness about the full range of income support programs, while another found newcomers were hesitant to access health and social services during the pandemic out of fear it would affect their residency status [48]. The interviewees echoed these concerns, stating that the lack of clear, understandable information made concerned communities “really nervous” and inhibited their access to services (4 C-KII).

CSOs introduced programs to fill information gaps identified by their constituents. These initiatives included translation services, including producing Indigenous language content about the pandemic in more than 100 different Indigenous languages and translating municipal guidelines (5 C-KII). Many included translators in workshops related to staying healthy or accessing services during the pandemic. Others relied on social media platforms like WhatsApp and Facebook (6 C-KII) and created website content in different languages (6 C-KII). Interviewees described how they considered religious and cultural beliefs when creating and sharing information, and targeted materials to meet specific populations, such as older adults. However, many CSO interviewees felt such contributions to policy implementation were not fully recognized by the government (3 C-KII).

#### Access to health services

13 policy documents identified focused on access to overall health services, including services for COVID-19 (vaccination services were categorized separately, see below). Multiple interviewees identified a policy gap in ensuring access to mental healthcare for priority populations (mentioned in eight policy documents). As one noted, “We know that stigma and discrimination against marginalized folks […] often increases when there’s kind of like social stress or like a public crisis. And so that among many other reasons, is why the need for mental health care spiked during the pandemic but people weren’t able to access” (2 C-KII). Barriers to accessing mental health care included costs, digital illiteracy as most mental health care was shifted to virtual delivery (7 C-KII), as well as labour policies, with newcomers and those precariously employed feeling unable to take time off to access care (3 C-KII).

Interviewees commented that access to healthcare was limited for many priority populations by lack of transportation options, as many people were hesitant to take public transit or ask others for rides due to physical distancing requirements. They noted that many Indigenous communities live in rural area where transportation is a particular barrier to accessing health services (5 C-KII). Certain demographics, such as older adults, individuals with disabilities, and immigrants, also often had limited mobility, making it harder for them to access health services. As one interviewee highlighted when speaking about immigrants trying to access testing and vaccination services, “. they wanted to, but they just couldn’t get there” (1 C-KII). Many priority populations faced other **cost** barriers to accessing health services during the pandemic (6 C-KII). Some newcomers did not have access to public health insurance, either because they had not been in the country long enough (wait periods differ by province but are often three months), or because they were undocumented [49]. The costs of access to health services were exacerbated by pandemic-induced job losses and increased financial insecurity [50].

#### Access to COVID-19 vaccinations

Out of the 70 identified policy documents, nine documents focused on vaccinations. The majority (*n* = 5) of these dealt with vaccination uptake especially among priority populations such as racialized groups, older adults, and Indigenous communities. Three documents outlined vaccination advice (e.g. on priority groups or booster vaccinations), and one document focused on standardized proof of vaccination. An interviewee reflected on the prioritization of population groups (speaking of older adults, racialized groups, Indigenous Peoples) by the government in its vaccine delivery strategy, indicating how the prioritization (i.e., equity focused programming and delivery of health services) served as a “catalyst” and aided in drawing attention to the needs of these particular population groups (7 C-KII).

Interviews highlight how priority populations faced challenges in physically accessing vaccines, due to lack of transportation (as described above and particularly in rural areas), lack of childcare support, and lack of support from their employer (1 C, 2 C, 3 C, 6 C -KII). One interviewee explained, “we had some community members express that they had unpaid or unavailable time off work to get vaccinated or they had a lack of childcare, so they had no one to take care of their child while they’re getting vaccinated, especially during the beginning. There were long lines. People had to wait like half a day, and it wasn’t something that they can afford to spend” (3 C-KII). These obstacles were compounded by time and resource costs, including difficulties in booking appointments, and barriers related to health issues, disabilities, and religious beliefs. Booking appointments was often hindered by complex and unfriendly online systems, as well as a lack of multilingual resources. Health issues and disabilities made physically accessing vaccination sites difficult for some.

Civil society organizations responded to the priority populations’ specific needs and developed interventions that included sharing information via appropriate channels, bringing vaccination services to the people, or supporting priority populations in accessing vaccination centers by providing transportation support (6 C-KII). Many also provided translation services. One CSO interviewed partnered with Public Health Agency of Canada to open multi-language COVID-19 vaccination clinic. Interpreters for different languages were available on specific days via the phone, video or in-person. A CSO-representative recommended “to make sure that every single clinic has somebody who– like they should have interpreters ready to go. And even if they’re not on site but be ready, you know, to call somebody at least on the phone.” (3 C-KII). CSO also helped priority populations access vaccines by providing transportation. An interviewee shared that “to address that we also had our community ambassadors go to apartments, to condos and they had vaccine clinics setup at the apartments” (1 C-KII). Another provided its constituents public transportation cards and ride‑sharing vouchers (6 C-KII).

## Discussion

This paper sought to examine the implementation of an equity-based policy responses to COVID-19, with the aim to inform future pandemic preparedness and response. It found that less than half (70/185) of federal policy documents analyzed included mention of priority populations or equity-related issues. When it came to implementation, the well-established GBA + framework and related existing relationships between government and CSOs created opportunities to put equity-based policies into action, while the urgency of the pandemic facilitated some unique opportunities for collaboration. However, this same urgency at times limited opportunities for consultations and reliance on CSOs to implement equity-based policies was limited by lack of resources [51]. Analysis of implementation gaps related to accessibility reveal how persistent contextual inequities– such as poor transportation networks - in access to information and resources impeded the ability of priority populations to benefit from equity-based pandemic policies. Equity-based policies gaps reflect uneven application of GBA+, urgency, lack of adequate consultation, and contextual constraints.

This analysis adds to the literature on GBA + implementation in Canada, as well as the growing literature on the limits of implementing equity-based policies more generally [52]. Substantial literature has also documented the fault lines within gender-mainstreaming (on which GBA + is based) including, limited consideration of intersecting inequities, the favoring approaches based on individual agency/behaviors, and lack of meaningful institutionalization [53]. This has included recent critiques of how gender considerations, in particular, have been integrated into pandemic preparedness and response globally, pointing out lack of meaningful consultation with women and priority populations, and the need to focus on equity during pandemic preparedness so as not to be reactionary [23, 54]. In this case, Canada’s long-standing commitment to GBA + provided a framework for integrating equity considerations by, for example, demanding any budget consideration conduct a GBA + analysis. The pre-pandemic establishment of government positions and units committed to GBA + meant there were people and resources capable of advancing equity considerations when the pandemic emerged. This speaks to the importance of establishing equity-based policy frameworks and relationships, as well as resources to support these, as a means to improve pandemic preparedness and mitigate risk of policy implementation gaps. However, federal actors also spoke of lack of time to meaningfully incorporate GBA + and CSOs spoke of tokenistic consultations. Such findings further point to how institutional constraints and practical challenges (such as those experienced by under resourced CSOs) to equity-based policy-making influence implementation gaps [55].

Such analysis further contributes to the literature on policy implementation gaps, particular in terms of considering how established conceptualization of implementation gaps apply to the pandemic policy context. For example, while dispersed government and inadequate collaboration are often cited as causes for policy gaps, this analysis suggests that pandemic policy making provided opportunities for closer relationships and collaborations. While implementation gaps during emergency response are often dismissed as a result of urgent timelines and multiple uncertainties [18], interviewees in this research spoke of creative opportunities for policy implementation, particularly related to the flexibilities created by unusual events (which in turn can justify unusual responses) and opportunities to circumvent politics or stringent processes. However, they also noted rapid timelines impeded evidence-based decision-making, perhaps leading to poorly designed policies. Bottom-up influences were the lack of adequate resources for the CSOs, which often acted as ‘street bureaucrat’ roles in implementing equity-based policies due to their established relationships with priority populations. While outside-in factors included the ability of priority populations to comply with policies due to contextual constraints reflected of long-standing structural inequities. Further robust research and analysis is needed on policy implementation and gaps during health crises in order to inform equity-based responses in future.

Our analysis of policy documents reveals concerted efforts to address the needs of a range of priority populations and equity issue areas, often in concert with each other, demonstrating an attempt to integrate intersectional approaches into pandemic response policy. The highest number of documents within our pool were issued from the Public Health Agency of Canada, demonstrating centralized leadership in implementing GBA+, with the support of select agencies such as Employment and Social Development Canada and the Indigenous Services Canada. The fact that other agencies, particularly those essential to ensuring pandemic response among priority populations such as Correctional Services Canada, had only a single equity-related policy, indicates uneven implementation. Priority populations most frequently mentioned represent those most affected by the pandemic, including older adults and racialized people. However, the lack of focus on other populations, such as those whose first language is not English, likely contributed to implementation gaps related to access to information, healthcare and vaccines. Indeed, accessibility was an overarching focus in the policy documents, and also emerged as a key theme related to implementation gaps during key informant interviews.

The focus on accessibility in policy documents suggest intentions to ensure priority populations did enjoy equitable access to health services and pandemic benefits. However, interviewees demonstrates that despite these intentions, neither information nor health services were easily accessible to many priority populations. Similarly, Lane and colleagues found that population groups, particularly newcomers, faced difficulties in understanding how and where to access care, and in navigating how the health system interacts with patients [56]. Kaufman et al. note that low uptake of pediatric vaccinations was influenced costs of transportation to get to appointments, challenges finding childcare for siblings, availability of vaccine appointments, and access to pertinent information regarding vaccines for their children [57]. Research further notes that reliable and trusted information is crucial to increase vaccine awareness and build trust and confidence in the government’s response to an emergency; yet CSOs reported that many newcomers could not access such information in their preferred language, and it was not always culturally appropriate [58, 59]. Many of these gaps reflected preexisting inequities in access to health information, transportation and resources that are shaped by broader economic, social and political systems, which were infrequently addressed in pandemic policies. For example, only two policy documents mentioned Indigenous peoples and colonization, reflecting a limited engagement with the structures that shape vulnerability.

The gaps revealed in accessible policies are particularly noteworthy as pandemic response relies heavily on the actions of the general population - and, for equity-based policies, priority populations within it - for implementation [10]. Barriers to information about the policies themselves inhibit priority populations from complying with policies, and barriers to access to healthcare and vaccination inhibit compliances with public health mandates. This suggests it was not the target groups’ preferences or beliefs that necessarily threatened policy implementation, but their lack of resources to undertake the behaviors prescribed. Notably, based on the perspectives of CSOs working with priority populations, possibilities to overcome these gaps were not complicated (finding transport, providing interpretation), but did require knowledge of the characteristics of populations and contexts of implementation, as well as funding or related resources.

This analysis is exploratory, relying on illustrative examples, and so has certain limitations and bounded by certain methodological constraints. The focus on federal policies, while enabling a national overview, does not consider the relationships with and policies implemented by Indigenous, provincial and regional governments. Our focus on policies directed at individuals or priority populations excluded essential workers (e.g., in transportation, retail, and agriculture), who often overlap with priority populations due to intersecting vulnerabilities. We also do not consider the effects of trust in government on policy implementation, an area of research gaining increasing attention as trust is a key determinant of willingness to comply with public health policies [60]. The decision to interview CSOs alongside federal actors means there is a notable focus on their participation in policy implementation, while other actors, such as healthcare providers and the private sector, are not included - again such an analysis requires further research. The sample of interviewees is small and therefore findings should not be considered generalizable but representative of select perspectives which, when read in relationship to existing literature, point to trends on policy implementation and gaps. Similarly, while we made extensive effort to source all federal policy documents that met our criteria not all policy decisions during the pandemic were documented and so might not be included here. Our inclusion criteria based on EDI glossary terms may also have missed documents that included equity considerations but did not use any of these terms. While our content analysis was verified through inter-author analysis and discussion, we recognize qualitative research necessarily includes an element of bias. We aimed to mitigate these limitations as much as possible through a reflexive and dialogical process that included ongoing discussions among authors.

## Conclusion

This research analyzes the attempts of the Canadian government to address the needs of priority populations during the COVID-19 pandemic. While our analysis focused on Canada, the findings provide insights into common implementation gaps and challenges in equity-based policies such as GBA+. The findings underscore the need for pre-established and intersectoral collaboration between the government and equity-focused organizations, as well as among various governmental departments or different CSOs, to ensure that future responses measures can be implemented swiftly while effectively reaching and supporting priority populations. For example, employing Indigenous knowledge systems and governance frameworks into pandemic responses has shown to enhance community resilience and trust [61]. Moreover, addressing systemic discrimination requires not only ongoing policy reform but also a transformation in how policies are developed and implemented. Equity-based approaches should move beyond consultation to co-designing strategies with affected communities, ensuring they are reflective of lived experiences and cultural contexts [62]. While addressing imbedded inequities requires ongoing policy efforts, rapid analysis of these inequities at the onset of a pandemic might inform strategies to mitigate.

## Electronic supplementary material

Below is the link to the electronic supplementary material.

Supplementary Material 1

Supplementary Material 2

## Data Availability

No datasets were generated or analysed during the current study.

## References

[1] Wenham C, Stout L. A legal mapping of 48 WHO member States’ inclusion of public health emergency of international concern, pandemic, and health emergency terminology within National emergency legislation in responding to health emergencies. Lancet. 2024;403(10435):1504–12. 10.1016/S0140-6736(24)00156-9 38527480 10.1016/S0140-6736(24)00156-9

[2] Haase A. Covid-19 as a social crisis and justice challenge for cities. Front Sociol. 2020;5:583638. 33869508 10.3389/fsoc.2020.583638 PMC8022683

[3] Tam T. From risk to resilience: an equity approach to COVID-19: the chief public health offficer of Canada’s report on the state of public health in Canada 2020. Ottawa: Government of Canada; 2020. https://www.canada.ca/content/dam/phac-aspc/documents/corporate/publications/chief-public-health-officer-reports-state-public-health-canada/from-risk-resilience-equity-approach-covid-19/cpho-covid-report-eng.pdf

[4] Paremoer L, Nandi S, Serag H, Baum F. Covid-19 pandemic and the social determinants of health. BMJ. 2021. 10.1136/bmj.n129 33509801 10.1136/bmj.n129 PMC7842257

[5] Schröders J, Wall S, Kusnanto H, Ng N. Millennium development goal four and child health inequities in Indonesia: A systematic review of the literature. PLoS ONE. 2015;10(5):e0123629. 10.1371/journal.pone.0123629.g001 25942491 10.1371/journal.pone.0123629 PMC4420469

[6] Burton ÉC, Bennett DH, Burton LM. COVID-19: health disparities and social determinants of health. Int Social Work. 2020;63(6):771–6. 10.1177/00208728

[7] Shadmi E, Chen Y, Dourado I, et al. Health equity and covid-19: global perspectives. Int J Equity Health. 2020;19(1). 10.1186/s12939-020-01218-z 10.1186/s12939-020-01218-z PMC7316580 32586388

[8] SWC (Status of Women Canada). GBA gender-based analysis plus. Demystifying GBA+: job aid. Ottawa: Status of Women Canada; 2017.

[9] Women and Gender Equality Canada. Government of Canada announces recipients of $100-million Feminist Response and Recovery Fund. February 11. 2021. https://www.canada.ca/en/women-gender-equality/news/2021/02/government-of-canada-to-invest-100-million-to-support-women-impacted-by-the-pandemic.html. Accessed April 2023.

[10] Kleinheksel AJ, Rockich-Winston N, Tawfik H, Wyatt TR. Demystifying content analysis. Am J Pharm Educ. 2020;84(1):7113. 10.5688/ajpe7113 32292185 10.5688/ajpe7113 PMC7055418

[11] Ansell C, Sørensen E, Torfing J. Improving policy implementation through collaborative policymaking. Policy Polit. 2017;45(3):467–86. 10.1332/030557317X14972799760260

[12] Raphael D. The political economy of health: a research agenda for addressing health inequalities in Canada. Can Public Policy. 2015;41:S17–25. 10.3138/cpp.2014-084

[13] McConnell A. Policy success, policy failure and grey areas in-between. J Public Policy. 2010;30(3):345– 62. Available from: http://www.jstor.org/stable/40925891. Accessed 16 Jul 2024.

[14] Hudson B, Hunter D, Peckham S. Policy failure and the policy-implementation Gap: can policy support programs help? Policy Des Pract. 2019;2(1):1–14. 10.1080/25741292.2018.1540378

[15] Pretorius L. Six contributions to understanding ‘gaps between policy and implementation’: An overview and comments. Politeia. 2003;22(1):6–21. Available from: https://hdl.handle.net/10520/EJC88083

[16] Kuhlmann S, Hellström M, Ramberg U, Reiter R. Tracing divergence in crisis governance: responses to the COVID-19 pandemic in France, Germany and Sweden compared. Int Rev Admin Sci. 2021;87(3):556–75. 10.1177/0020852320979359

[17] Benvenisti E. The WHO– Destined to Fail? Political Cooperation and the COVID-19 Pandemic (June 30, 2020). University of Cambridge Faculty of Law Research Paper No. 24/2020. 10.2139/ssrn.3638948

[18] Lancaster K, Rhodes T, Rosengarten M. Making evidence and policy in public health emergencies: lessons from COVID-19 for adaptive evidence-making and intervention. Evid Policy. 2020;16(3):477–90. 10.1332/174426420X15913559981103

[19] Mason DJ, Perez A, McLemore MR, Dickson E. Policy & politics in nursing and health care. 7th ed. [E-book]. Elsevier Health Sciences; 2020 Jan 28. Available from: https://books.google.ca/books?id=NGGuCAAAQBAJ

[20] Government of Canada. Government of Canada’s approach on Gender-based Analysis Plus. [cited 2024 Jul 16]. Available from: https://www.canada.ca/en/women-gender-equality/gender-based-analysis-plus/government-approach.html

[21] Women and Gender Equality Canada. Action Plan on Gender-based Analysis (2016–2020). https://women-gender-equality.canada.ca/en/gender-based-analysis-plus/resources/action-plan-2016-2020.html. Published April 2021. Accessed July 2024.

[22] Government of Canada Justice Laws. Canadian Gender Budgeting Act. S.C 2018, C. 27, S.314. Accessed April 10, 2023. https://laws-lois.justice.gc.ca/PDF/C-17.2.pdf

[23] Smith J, Mũrage A, Lui I, Morgan R. Integrating gender-based analysis plus into policy responses to COVID-19: lived experiences of lockdown in British Columbia, Canada. Soc Polit. 2022;29(4):1168–91. 10.1093/sp/jxac024 36533213 10.1093/sp/jxac024 PMC9755975

[24] Tikkanen R, Osborn R, Mossialos E, Djordjevic A, Wharton GA. International health care system profiles - Canada. New York: The Commonwealth Fund; [cited 2024 Jul 16]. Available from: https://www.commonwealthfund.org/international-health-policy-center/countries/canada

[25] Macdonald D. Still picking up the tab: federal and provincial government COVID-19 spending. Ottawa: Canadian Centre for Policy Alternatives; 2021 Aug [cited 2024 Jul 16]. Available from: https://policyalternatives.ca/sites/default/files/uploads/publications/National Office/2021/08/Still picking up the tab.pdf

[26] Macdonald D. Picking up the tab: A complete accounting of federal and provincial government COVID-19 measures in 2020. Ottawa: Canadian Centre for Policy Alternatives; 2020 [cited 2024 Jul 16]. Available from: https://policyalternatives.ca/sites/default/files/uploads/publications/National Office/2021/01/Picking up the tab.pdf

[27] Government of Canada. Guide on equity, diversity and inclusion terminology. [cited 2024 Jul 16]. Available from: https://www.noslangues-ourlanguages.gc.ca/en/publications/equite-diversite-inclusion-equity-diversity-inclusion-eng

[28] British Columbia Centre for Disease Control. BCCDC COVID-19 language guide: guidelines for inclusive language for written and digital content. [cited 2024 Jul 16]. Available from: http://www.bccdc.ca/Health-Professionals-Site/Documents/public_health/Language_Guide.pdf

[29] National Collaborating Centre for Determinants of Health. Glossary of essential health equity terms. Antigonish (NS): NCCDH, St. Francis Xavier University; [updated 2022; cited 2024 Jul 15]. Available from: https://nccdh.ca/learn/glossary/

[30] Parkinson S, Eatough V, Holmes J, Stapley E, Target M, Midgley N. Framework analysis: a worked example of a study exploring young people’s experiences of depression. Qual Res Psychol. 2016;13(2):109– 29. Available from: https://eprints.bbk.ac.uk/id/eprint/13389/2/13389.pdf

[31] Hennink MM, Kaiser BN, Marconi VC. Code saturation versus meaning saturation: how many interviews are enough? Qual Health Res. 2017;27(4):591–608. 10.1177/1049732316665344 27670770 10.1177/1049732316665344 PMC9359070

[32] Fryer S, Leblanc-Laurendeau O. Understanding federal jurisdiction and First Nations. Ottawa: Library of Parliament; 2021 [cited 2024 Jul 16]. Available from: https://lop.parl.ca/sites/PublicWebsite/default/en_CA/ResearchPublications/201951E

[33] Government of Canada. Government of Canada invests more than $28 million to support the funding of virtual health care services in Quebec. Ottawa, Ontario: Health Canada; 2021 Dec 16 [cited 2024 Jul 16]. Available from: https://www.canada.ca/en/health-canada/news/2021/12/government-of-canada-invests-more-than-28-million-to-support-the-funding-of-virtual-health-care-services-in-quebec.html

[34] Office of the Prime Minister. Prime Minister announces health and social support for northern communities. Ottawa: Government of Canada; 2020 [cited 2024 Jul 16]. Available from: https://www.pm.gc.ca/en/news/news-releases/2020/04/14/prime-minister-announces-health-and-social-support-northern

[35] Indigenous Services Canada. Indigenous health management and initiatives. Ottawa: Government of Canada. 2020 [cited 2024 Jul 16]. Available from: https://www.sac-isc.gc.ca/eng/1581897443592/1581897469233

[36] Greenwood M, de Leeuw S, Lindsay N, Reading C, editors. Determinants of Indigenous peoples’ health in Canada: beyond the social. Toronto, ON: Canadian Scholars’; 2015.

[37] Greenwood M, de Leeuw S, Lindsay N, Reading C. Challenges in health equity for Indigenous peoples in Canada. Lancet. 2018;391:1645–47. 29483024 10.1016/S0140-6736(18)30177-6

[38] Canadian Heritage. Building a more inclusive Canada: Government of Canada announces funding for anti-racism projects across the country. Ottawa: Government of Canada; 2020 [cited 2024 Jul 16]. Available from: https://www.canada.ca/en/canadian-heritage/news/2020/10/building-a-more-inclusive-canada-government-of-canada-announces-funding-for-anti-racism-projects-across-the-country.html

[39] Crenshaw K. Mapping the Margins: Intersectionality, Identity Politics, and Violence against Women of Color. Stanford Law Review, 1991;43(6):1241–99. JSTOR, 10.2307/1229039. Accessed 15 Dec. 2024.

[40] Government of Canada. Government of Canada funds two new projects to encourage COVID-19 vaccination in youth. Brampton, Ontario: Public Health Agency of Canada; 2021 Jul 27 [cited 2024 Jul 16]. Available from: https://www.canada.ca/en/public-health/news/2021/07/government-of-canada-funds-two-new-projects-to-encourage-covid-19-vaccination-in-youth.html

[41] Indigenous Services Canada. Government of Canada is providing assistance to urban Indigenous organizations in the Greater Toronto Area to address the COVID-19 pandemic. Ottawa: Government of Canada; 2020 [cited 2024 Jul 16]. Available from: https://www.canada.ca/en/indigenous-services-canada/news/2020/08/government-of-canada-is-providing-assistance-to-urban-indigenous-organizations-in-the-greater-toronto-area-to-address-the-covid-19-pandemic.html

[42] Employment and Social Development Canada. Emergency Community Support Fund. Ottawa: Government of Canada; 2020 [cited 2024 Jul 16]. Available from: https://www.canada.ca/en/services/benefits/emergency-community-support-fund.html

[43] Canadian Heritage. Statistics on official languages in Canada. Ottawa: Government of Canada; 2024 [cited 2024 Jul 16]. Available from: https://www.canada.ca/en/canadian-heritage/services/official-languages-bilingualism/publications/statistics.html

[44] Public Health Agency of Canada. Immunization Partnership Fund: Increasing confidence, acceptance and uptake of Covid-19 vaccines. Ottawa: Government of Canada; 2021 [cited 2024 Jul 16]. Available from: https://www.canada.ca/en/public-health/services/funding-opportunities/grant-contribution-funding-opportunities/immunization-partnership-fund-increasing-confidence-acceptance-uptake-covid-19-vaccines.html

[45] Indigenous Services Canada. Accessing COVID-19 public health support for First Nations communities. Ottawa: Government of Canada. 2023 [cited 2024 Jul 16]. Available from: https://www.sac-isc.gc.ca/eng/1584819394157/1584819418553#b

[46] GoC Communications Community Office. Plain language, accessibility, and inclusive communications. Ottawa: Government of Canada; 2024 [cited 2024 Jul 16]. Available from: https://www.canada.ca/en/treasury-board-secretariat/topics/government-communications/communications-community-office/communications-101-boot-camp-canadian-public-servants/plain-language-accessibility-inclusive-communications.html

[47] Public Health Agency of Canada. Government of Canada fund new project to encourage vaccine uptake in Canada. Ottawa: Government of Canada; 2021 [cited 2024 Jul 16]. Available from: https://www.canada.ca/en/public-health/news/2021/06/government-of-canada-funds-new-project-to-encourage-vaccine-uptake-in-canada.html

[48] Mũrage A, Smith J. Multifaceted precarity: pandemic experiences of recent immigrant women in the accommodation and food services sector. BMC Public Health. 2023;23(1):2497. 10.1186/s12889-023-17392-y 38093212 10.1186/s12889-023-17392-y PMC10716935

[49] Hamel-Smith Grassby M, Wiedmeyer ML, Lavergne MR, Goldenberg SM. Qualitative evaluation of a mandatory health insurance ‘wait period’ in a publicly funded health system: understanding health inequities for newcomer im/migrant women. BMJ Open. 2021;11(8). 10.1136/bmjopen-2020-047597 10.1136/bmjopen-2020-047597 PMC8344307 34353797

[50] Tan SY, Foo C, Verma M, et al. Mitigating the impacts of the COVID-19 pandemic on vulnerable populations: lessons for improving health and social equity. Soc Sci Med. 2023;328:116007. 10.1016/j.socscimed.2023.116007 37279639 10.1016/j.socscimed.2023.116007 PMC10234832

[51] Public Health Agency of Canada. Government of Canada funds five new projects to encourage COVID-19 vaccination in Canada. Ottawa, Ontario: Government of Canada. 2021 Jul 12 [cited 2024 Jul 16]. Available from: https://www.canada.ca/en/public-health/news/2021/07/government-of-canada-funds-five-new-projects-to-encourage-covid-19-vaccination-in-canada.html

[52] Meier P, Celis K. Sowing the seeds of its own failure: implementing the concept of gender mainstreaming. Soc Polit. 2011;18(4):469–89. 10.1093/sp/jxr020

[53] Moser C, Moser A. Gender mainstreaming since Beijing: a review of success and limitations in international institutions. Gend Dev. 2005;13(2):11–22. 10.1080/13552070512331332283

[54] Harman S. Threat not solution: gender, global health security and COVID-19. Int Aff. 2021;97(3):601–23. 10.1093/ia/iiab012

[55] Hoppe R. Institutional constraints and practical problems in deliberative and participatory policy making. Policy Politics. 2011;39:2. 10.1332/030557310X519650. accessed Dec 15, 2024.

[56] Lane G, Hengstermann M, White J, Vatanparast H. Newcomer challenges with accessing healthcare services in Saskatchewan, Canada. Bord Cross. 2021;11(2):155–72. 10.33182/bc.v11i2.1222

[57] Kaufman J, Tuckerman J, Bonner C, Durrheim DN, Costa D, Trevena L, Thomas S, Danchin M. Parent-level barriers to uptake of childhood vaccination: a global overview of systematic reviews. BMJ Global Health. 2021;6(9). 10.1136/bmjgh-2021-006860 10.1136/bmjgh-2021-006860 PMC8477248 34580071

[58] Schluter AP, Généreux M, Landaverde E, Schluter PJ. In the COVID-19 pandemic, who did we trust? An eight-country cross-sectional study. J Glob Health. 2023;13:06036. 10.7189/jogh.13.06036 37651637 10.7189/jogh.13.06036 PMC10471152

[59] Ji C, Jiang J, Zhang Y. Political trust and government performance in the time of COVID-19. World Dev. 2024;176:106499. 10.1016/j.worlddev.2023.106499

[60] Schluter AP, Généreux M, Landaverde E, Schluter PJ. In the COVID-19 pandemic, who did we trust? An eight-country cross-sectional study. J Glob Health. 2023;13:06036. 10.7189/jogh.13.06036. PMID. 10.7189/jogh.13.06036 PMC10471152 37651637

[61] Mzimela JH, Moyo I. On the efficacy of Indigenous knowledge systems in responding to the COVID-19 pandemic: unsettling coloniality. Int J Environ Res Public Health. 2024;21(6):731. 10.3390/ijerph21060731 38928977 10.3390/ijerph21060731 PMC11203967

[62] Bartlett A, Faber S, Williams M, Saxberg K. Getting to the root of the problem: supporting clients with Lived-Experiences of systemic discrimination. Chronic Stress (Thousand Oaks). 2022;6:24705470221139205. 10.1177/24705470221139205 36439647 10.1177/24705470221139205 PMC9685113

