# Supplementary material for: “They wanted to, but they just couldn’t get there”: GBA + implementation and gaps during the COVID-19 pandemic in Canada
Source: Int J Equity Health. 2025 May 27;24:152. doi: 10.1186/s12939-025-02522-2 (PMC12117922; doi:10.1186/s12939-025-02522-2)
Supplement: Supplementary file 1 — Supplementary Material 1 [file 12939_2025_2522_MOESM1_ESM.docx]

***Policies included in the Analysis***

Economic:

| **Policy** | **Date (Month Year)** | **Priority Populations** | **Issue Areas** |
| --- | --- | --- | --- |
| **Canada Emergency Student Benefit** | May 2020 | Students (incl. students with disabilities)  People with low income | - Accessibility |
| **Indigenous Community Support Fund** | May 2020 | Indigenous, First Nation, Metis, and Inuit communities and organizations | - Mental Health |
| **GST/ HST Credit** | July 2020 | People with low income | - Accessibility |
| **Childcare Benefit** | July 2020 | Families and children | - Accessibility |
| **One-time, tax-free payment** | July 2020 | People with low income  Older adults | - Ageism |
| **One-time payment for persons with disabilities** | July 2020 | People with disabilities | - Accessibility |
| **Funding for Urban Indigenous Organizations** (GTA) | August 2020 | Indigenous, First Nation, Metis, and Inuit communities and organizations | - Accessibility |
| **Expanded Employment Insurance (EI) Program** | September 2020 | People with disabilities  People with low-income  Families  Children  Unpaid caregivers  People with employment barriers | - Accessibility |
| **COVID-19 Measures for Students and Young Canadians** | October 2020 | People with low-income  Youth  Students  People with employment barriers | - Diversity, - Disability |
| **Canada Recovery Caregiving Benefit** | October 2020 | Families Children/ youth People with low income People with employment barriers Caregivers | - Accessibility |
| **Canada Recovery Sickness Benefit** | October 2020 | People with low income  People with employment barriers | - Accessibility |
| **Canada Child Benefit** | December 2020 | Families Children/ youth People with low income |  |
| **Canada Recovery Sickness Benefit and Caregiving** (ext.) | February 2021 | Families Children/ youth People with low income Caregivers People with employment barriers | - Equity |
| **Canada Emergency Wage Subsidy and Rent Subsidy** (ext.) | February 2021 | People with low income  People with employment barriers | - Accessibility - Equity |
| **Employment Insurance Program Expansion** | March 2021 | People with low income  People with employment barriers | - Accessibility |
| **2021 Budget "A Recovery Plan for Jobs, Growth, and Resilience"** | April 2021 | Aboriginal, Black, Inuit, LGBTQ2, First Nation, Gender, Metis, Racialized pops. | - Accessibility - Ableism - Ageism - Colonialism - Colonization - Disability - Discrimination - Diversity - Equity - Equality - Ethnic - GBA+ - Homophobia - Indigenous - Intersectionality - Marginalized - Minority - Prejudice - Racism - Sexism - Transphobia - Trauma - White - Reconciliation - Under-represented |
| **Funding for the Prince George Nechako Aboriginal Employment and Training Association** | May 2021 | Indigenous, First Nation, Metis, and Inuit communities and organizations | - Mental health, - Diversity |
| **One-time payment for low-income seniors** | February 2022 | Older adults with low income |  |

Health:

| **Policy** | **Date (Month Year)** | **Priority Populations** | **Issue Areas** |
| --- | --- | --- | --- |
| Vaccine | | |  |
| **Priority Population for Vaccination** | Dec 2020 | Older Adults, Indigenous, HCWs | Equity, Ageism |
| **Investment of $ 2.25 million for 2 projects, ScienceUp First and CARD (C-Comfort, A-Ask, R-Relax, D-Distract) System, to support vaccination efforts** | April 2021 | No population specified. | - Accessibility - Equity - Equality - Marginalized |
| **$25,000 awarded to 20 finalists participating in the Vaccine Community Innovation Challenge** | June 2021 | People with Disabilities, low-income, older adults, racialized people (Black, BIPOC), Indigenous, First Nations | - Intersectionality - Accessibility - Ethnicity |
| **Funding of 5 projects (4 provincial and 1 national) through the Immunization Partnership Fund – Inc. vaccine knowledge & access** | June 2021 | No population specified. | Equity, Equality |
| **funding of 4 projects through the Immunization Partnership Fund –** *Inc. uptake among disproportionate pops via culturally relevant interventions* | June 2021 | Women, Newcomer, Racialized person (black) | Gendered impacts, Ethnicity, First Language |
| **funding of 2 projects through the Immunization Partnership Fund** *– Inc. vaccine promotion among youths* | June 2021 | Youths | Equity, Accessibility |
| **Vaccination of 600 older, medically vulnerable federal inmates** | August 2021 | Older adults | Accessibility |
| **Standardization of COVID-19 proof of vaccination for all provinces and territories** | October 2021 | Immigrant, Newcomer, Indigenous | Equality, Equity, Accessibility |
| **New recommendations for booster** | April 2022 | Indigenous, Inuit, First Nations, Older adults | Accessibility |
| Health workforce | | |  |
| **$50 million to support purchase of PPE for provinces & address federal needs** | March 2020 | First Nations, Indigenous, Inuit | - Accessibility, - Equity, - Marginalization |
| **Canadian Armed Forces reserves, will be deploying to Inuit communities of northern Quebec to assist with the public health response to COVID-19** | April 2020 | First Nations, Inuit | - Accessibility, - Remote |
| **Canadian Armed Forces deployed to provide support to Ontario long-term care facilities.** | April 2020 | Older adults | - Accessibility |
| **Canadian Ranger Patrol group deployed 200 rangers, in response to various requests for assistance from Quebec** | April 2020 | First Nations, Indigenous | - Remote, - Accessibility, - Colonialism |
| **$285.1 million to support the ongoing public health response to COVID-19 in Indigenous communities** | May 2020 | First Nations, Metis, Inuit, LGBTQ | - Accessibility - Equity - Equality - Intersectionality (violence prevention projects for Métis women, girls, and LGBTQ and two-spirit people) |
| **Funding the Canadian Red Cross to rapidly scale up a new humanitarian workforce to complement existing health measures in long-term care and residential facilities** | July 2020 | Older adults | - Accessibility - Ageism |
| **Creation of Essential Services Contingency Reserve that complements existing PPE support being provided to front-line health care workers** | July 2020 | Women, Racialized persons | - Privilege - Marginalization |
| **Deployment of federal health human resources (CAF & redeployment of the CRC) to augment or relieve staff within medical care facilities in Ontario.** | April 2021 | Older adults | - Ageism - Accessibility - Equality |
| **$70 million funding to the Canadian Red Cross to build and maintain capacity to support deployment for crisis management activities and for COVID-19 testing-related activities** | April 2021 | Older adults, Racialized persons | - Equity - Accessibility - Remote |
| **Canadian Rangers and the CAF were deployed to aid with COVID-19 response efforts in the Moose Cree First Nation and the Neskantaga.** | May 2021 | Indigenous, First Nation, Inuit, Metis | - Equity - Remote |
| **Assistance in the form of CAF Rangers to support vaccination campaign in remote communities in northern Ontario** | November 2021 | Indigenous, First Nation, Inuit, Metis | - Equity - Remote |
| **Amendment to Canada's Criminal Code to enhance protection of health care workers, those who assist them and those accessing health care service.** | December 2021 | Women, Racialized person | - Gender - Discrimination |
| **Investment of $379 million into long-term care in Ontario** | April 2022 | Older adults | - Equity - Accessibility |
| Health Communication | | |  |
| **Launched a web-based email service that provides subscribers with critical information related to the pandemic** | April 2020 | No specific population. | - Remote - Marginalization - Accessibility |
| **Launched the "Ask the Experts" campaign series to answer common questions about COVID-19 vaccines** | June 2021 | Women, Youths, Ethnicity, Race, Newcomers | - Minority - Accessibility |
| **Allocated funding to the Dr. Peter Centre to develop a bilingual national community of practice** | June 2021 | Immigrants, Newcomers, PwD, People whose 1^st^ language is not English. | - First language - Accessibility - Equity - Disability |
| Services | | |  |
| **Investment of $9M to support practical services to Canadian seniors by local organization through United Way Canada** | March 2020 | Older adults, low-income, women, youths, Gender | - Ageism, - Marginalized |
| **$7.5M in funding to Kids Help Phone to provide children and youth with mental health support.** | March 2020 | Youths | - Mental health |
| **$157.5 million to address the needs of Canadians experiencing homelessness through the Reaching Home program** | April 2020 | Low-income, homelessness | - Equality - Equity - safety |
| **Shipped 275 orders for personal protective equipment including hand sanitizer, N95 masks, isolation shields and gloves to Indigenous communities** | April 2020 | Indigenous, First Nations, Metis | - Equity - Accessibility |
| **Additional support to address immediate health, economic and transportation needs in the North (governments of Yukon, the Northwest Territories and Nunavut** | April 2020 | Indigenous | - Accessibility - Remote |
| **Expanded pharmacist policies and benefit coverage for First Nations and Inuit clients as part of the Non-Insured Health Benefits program** | April 2020 | Indigenous, First Nations | - Accessibility |
| **Expanded dentist and audiologist policies and benefit coverage for First Nations and Inuit clients for virtual health services as part of the Non-Insured Health Benefits program** | April 2020 | Indigenous, Inuit | - Accessibility |
| **Investment of $240.5 million to develop, expand and launch virtual care and mental health tools to support Canadians** | May 2020 | No population specified. | - Mental health - Accessibility - Marginalized |
| **Indigenous Mental Wellness Support - $82.5 million in mental health and wellness supports to help Indigenous communities** | August 2020 | Indigenous, First Nations | - Mental health |
| **Investment of $11.5 million in mental health funding for organizations, and to tackle systemic challenges and barriers, including those faced by Black Canadians.** | September 2020 | Racialized persons, Black | - Mental health |
| **$186.8 million over 2 years to support the needs and gaps in long-term care facilities, and to provide additional home care in Indigenous communities** | January 2021 | Older adults, Indigenous, Inuit, Metis | - Equity, Equality |
| **Support mental health programs for Black LGBTQI+ Canadians - investment of up to $1.28 million in funding for three organizations** | September 2021 | Black, LGBTQ | - Intersectionality (mental health, substance use & addiction, STTBBI interventions for black LGBTQ people) - Mental health |
| **Canada-Quebec agreement for virtual health care services in the context of the COVID-19 pandemic** | December 2021 | No population specified. | - Accessibility - Marginalized - Remote |

Public Safety:

| **Policy** | **Date (Month Year)** | **Priority Populations** | **Issue Areas** |
| --- | --- | --- | --- |
| **$40 million to support violence against women shelters & sexual assault centres + $10 million to ISC to support shelters in Yukon** | April 2020 | Indigenous, First nations, Gender, Youths | Trauma, Safety |
| **$10 million in funding being distributed to nearly 1000 organizations that provide important services to those experiencing gender-based violence** | July 2020 | Women | Trauma, Safety |
| **Providing $15 million for 85 anti-racism projects across the country** | October 2020 | Racialized person, People with disability, Indigenous, Religious minority, Gender | - Racism - Discrimination - Sexual orientation - Minority |
| **Investing $350 million to help community organizations serve vulnerable Canadians during the COVID-19 crisis.** | October 2020 | No population specified. (‘Vulnerable’ term used only)  Org. website mention: LGBTQ, women, race, Indigenous | Marginalized |
| **Creation of an Advisory Panel on systemic racism, discrimination+ for Dept of National Defence & CAF** | December 2020 | Indigenous, Racialized person (Black/Poc), Women, LGBTQ | - Racism - Gender - White supremacy - Minority - Sexual orientation |
| **Investment of $9,275,000 in 57 distress centres, to support a range of distress centres across the country, including in rural and remote area** | June 2021 | Older adults, Indigenous, LGBTQ, Racialized persons, people whose 1^st^ language isn’t English | - Mental health - First language - Ageism |
| **Release of ‘the 2021 Federal Pathway to Address Missing and Murdered Indigenous Women, Girls, and 2SLGBTQQIA+ People’ – 1st Annual Progress Report** | June 2021 | Indigenous, Metis, Inuit, First Nations women (Gender) | Trauma, Safety |
| **Launch of Task Force to conduct the most extensive review of the Employment Equity Act since its introduction.** | July 2021 | Women/Gender, Racialized person, Black, LGBTQ, People with Disabilities | - Disability - Race - Equality |
| **Communities at Risk: Security Infrastructure Program (SIP) - Funding is available to private, not-for-profit organizations that are at risk of being victimized by hate- motivated crime.** | October 2021 | Racialized person, Newcomer, Religious minority, LGBTQ | - Race - Sexual orientation - Ethnicity |
| **Promise of $250 million over five years, from 2021-22, for municipalities and Indigenous communities to support community-based prevention and intervention programs to reduce gun and gang violence.** | December 2021 | Indigenous youths and their families | - Safety |
| **Dept of National Defence report released that provides recommendations for change in thirteen areas across the organization** | April 2022 | Indigenous, LGBTQ, Racialized persons (PoC, Black), Women/Gender | - Racism - Reconciliation - Gender |
| **$682K for 2 orgs based in BC, focused on ending gender-based violence and negative stereotypes towards Indigenous women** | December 2022 | Women/Gender | - Trauma - Safety |
| **Community Support, Multiculturalism, and Anti-Racism Initiatives Program** | December 2022 | Indigenous, Racialized person | - Racism, - Discrimination, - Diversity |
| **Foundation for Black Communities was selected to enter negotiations for the administration of the $200 million Black-led Philanthropic Endowment Fund.** | February 2023 | Racialized person (Black) | - Equity, - Racism, - Diversity |
