# Supplementary material for: “They wanted to, but they just couldn’t get there”: GBA + implementation and gaps during the COVID-19 pandemic in Canada
Source: Int J Equity Health. 2025 May 27;24:152. doi: 10.1186/s12939-025-02522-2 (PMC12117922; doi:10.1186/s12939-025-02522-2)
Supplement: Supplementary file 2 — Supplementary Material 2 [file 12939_2025_2522_MOESM2_ESM.docx]

**Draft Interview Guide**

**for Key Informant Interviews**

1. Can you tell us a bit about your role:

- About the position you hold, your tasks, and about the organization etc?
- What is your organizational focus, reach, and scope?

1. In your opinion and/or experience, how does your organization understand and seek to advance health equity?
2. How was this understanding/approach applied during the COVID-19 pandemic?

- Have you focused on any particular population or issue areas? In what ways? Why?
- Can you describe your experience in working with these priority populations during this public health crisis in your role?
- What have been the successes and what challenges?
- Did you work with any other partners -Why? And what form did that collaboration take?

1. Does your organization have any pre-existing emergency/crisis response policies or guidelines that informed its pandemic response initiatives?

- If yes, in what ways was it applied during COVID? In what ways was it helpful or not? Were any policy documents used to inform your own response?
- If not, do you think such guidance would be helpful? What would you want it to include?

1. Based on your experiences in the role, what should be preferred approaches of consultation from health decision/policy-makers during health crises?
2. What recommendations would you make to other civil society organizations or policymakers in incorporating equity and intersectionality in pandemic preparedness planning and response?
3. What are your perspectives on the most appropriate data or indicators to inform monitoring of pandemic preparedness? Please elaborate.

1. Do you have anything further you would like to share?
